# To the Profession

**Published:** 1874-05

**Authors:** G. W. Keely, G. F. S. Wright, J. N. Crouse

**Affiliations:** Oxford, Butler County, Ohio; Pomaria, South Carolina; 652 Wabash Ave., Chicago, Ill.


### TO THE PROFESSION.

We trust the profession will give special attention to the following circular, not only in the way of reading it once or twice and think how good a thing Rubber Dam is, but make a demonstration of another kind. Let this committee hear from every dentist in the United States who uses Rubber Dam to the extent of $5 to $20.

No one need feel limited even to the latter amount. We can hardly see how any one can sleep well after having used this invaluable appliance and received the advantages which it gives, without making some acknowledgment of the fact to him who devised and presented it to the profession. A boon to the profession and to the people, the value of which is beyond all estimate.

Then let every body contribute something to the small acknowledgment which the profession proposes to make to Dr. Barnum.

We would respectfully suggest that every dental journal in the country publish this circular.

To The Dental Profession.

At the last meeting of the "American Dental Association" at Put-in-Bay, the following resolution was offered:

"Resolved, That a committee of three be appointed to carry into effect the resolution passed at Nashville, Tennessee, viz: To raise one thousand dollars to present Dr. S. C. Barnum, as a slight appreciation of his invaluable gift to the profession."

This resolution was unanimously adopted, and the following committee appointed by the President: G. W. Keely, Ohio; G. F. S. Wright, South Carolina; G. N. Crouse, Illinois; [see page 22 last transactions.]

It is well known that Dr. Barnum generously donated the use of the Rubber Dam to the profession, now so indispensable in the operation of filling teeth, and it is universally acknowledged to be one of the most important appliances in the hands of the Dentist, so much so, that no operator can afford to do without it.

The committee feel, in presenting this claim, they are but giving the blessed privilege of contributing to this testimonial fund, and hope to increase it, to ten times the amount contemplated in the resolution. Dr. Barnum has never asked anything of the profession, had he secured a patent, and charged each dentist a moderate sum for its use, to-day he might have been wealthy. No dentist, who does any considerable amount of operating would do without the Rubber Dam, even if it cost him one hundred dollars per year.

Now let us do our friend justice, by at once giving our mite to this fund, that our conscience may be at rest. As the time is short before the committee are to report at Detroit, Michigan, first Tuesday in August, it is important that contributions be sent at once, to either of the committee, with an expression of opinion as to the value of the Rubber Dam, that these letters may be preserved and presented to Dr. Barnum with the fund.

The committee would also suggest the propriety of calling meetings in all cities, to take action and assist in this good work, and societies contribute from their funds, which can be sent now, or handed to them at Detroit, the latter would be preferred. But one society has been directly appealed to, and all the money in the Treasury was freely given. The committee desire that all funds raised, be applied directly to the object, and to avoid expense, would respectfully ask the assistance and co-operation of the profession in this good work. All funds sent us should be by draft or post-office money order.

When any considerable amount is sent, let it be by draft on New York, payable to the order of S. C. Barnum.

G. W. Keely, Oxford, Butler County, Ohio.
G. F. S. Wright, Pomaria, South Carolina.
J. N. Crouse, 652 Wabash Ave., Chicago, Ill.

*Oxford, Butler County, Ohio.*
*April*, 1874.

